# The relative voltage index: a novel tailored method to identify left atrial low voltage areas in non-paroxysmal AF

**DOI:** 10.3389/fcvm.2025.1656983

**Published:** 2025-09-16

**Authors:** Guoshu Yang, Shiqiang Xiong, Yan Luo, Duan Luo, Michael Shehata, Zhen Zhang, Lin Cai, Xunzhang Wang, Ashkan Ehdaie, Hanxiong Liu

**Affiliations:** ^1^ Department of Cardiology, Affiliated Hospital of Southwest Jiaotong University, The Third People’s Hospital of Chengdu, Chengdu, Sichuan, China; ^2^ Smidt Heart Institute, Cedars Sinai Medical Center, Los Angeles, CA, United States

**Keywords:** atrial fibrillation, electroanatomic mapping, fibrosis, low voltage areas, left atrium, left atrial appendage, magnetic resonance imaging

## Abstract

**Background:**

The optimal voltage threshold for determining low voltage areas (LVA) in non-paroxysmal atrial fibrillation (*N*PAF) is unclear. This study aims to evaluate a patient-specific voltage measurement using the left atrial appendage (LAA) as a benchmark to establish a normalized threshold for detecting LVA in NPAF.

**Methods:**

Bipolar LA and LAA voltage in 40 NPAF and 42 patients with no AF (control group) were studied in sinus rhythm (SR) and AF (NPAF group) and SR only in the control group. Bipolar LA and LAA voltage distribution were compared between the NPAF and control groups. Fibrotic regions identified by late gadolinium-enhanced magnetic resonance imaging (LGE-MRI) was used as the reference standard comparison in the NPAF group.

**Results:**

The median, 5th percentile (V_LA5%_), and the 95th percentile of bipolar voltage in the LA were significantly lower in NPAF patients than controls. No significant LAA voltage differences between groups [median = 3.303 (1.796) vs. 3.100 (1.045); V_LAA95%_ = 8.089 (3.571) vs. 7.604 (3.404), all *p* > 0.05]. A strong linear correlation between V_LA5%_ and V_LAA95%_ was observed in the control group. The standardized relative voltage index (RVI) factor of 0.1324 was identified as the threshold for defining LVA and calculated as V_LA5%_ = 0.1324 × V_LAA95%_. The correlation between LVA guided by RVI was superior to the universal threshold for detecting LVA in sinus and AF rhythms using LGE-MRI as the gold standard.

**Conclusion:**

A patient-tailored low voltage threshold can be obtained using a simplified equation and provides more accurate representation of LVA in NPAF than universal thresholds.

## Introduction

Atrial fibrillation (AF), the most common cardiac arrhythmia, significantly contributes to increased risks of stroke, heart failure, and mortality (1, 2). Fibrosis of the left atrium (LA) represents a well-established pathological substrate that promotes both initiation and maintenance of AF (3). Notably, non-paroxysmal AF (NPAF) demonstrates greater fibrotic burden compared to paroxysmal AF (PAF) (4). This structural remodeling can be non-invasively characterized through late gadolinium-enhanced magnetic resonance imaging (LGE-MRI), while electroanatomic mapping reveals corresponding low-voltage areas (LVA) (5–7). Substantial evidence confirms a robust correlation between LVA and atrial fibrosis in AF (8–12). Furthermore, the presence of LVA has been demonstrated as a significant predictor of AF recurrence following catheter ablation (13).

Although adjunctive LVA ablation has been proposed for NPAF substrate modification, clinical outcomes remain inconsistent (14). This variability may stem from non-standardized, patient-nonspecific LVA definitions. Thus, determining optimal voltage thresholds for clinically relevant LVA ablation targets in NPAF remains an area of ongoing research and debate. To address this knowledge gap, the aim of this study was to evaluate the feasibility of utilizing patient-specific voltage measurements from the left atrial appendage (LAA) as an intrinsic reference to establish a normalized LVA detection threshold in NPAF.

## Materials & methods

### Study population

A total of 82 patients were enrolled in the study between April 2017 and August 2022. The NPAF group included 40 patients (24 with persistent AF and 16 with long-standing persistent AF) who underwent initial radiofrequency catheter ablation (RFCA). The control group consisted of 42 patients with structurally normal hearts and no previous history of AF who underwent left-sided catheter ablation procedures [*N* = 35 left-sided accessory pathway, *N* = 7 idiopathic ventricular premature contractions (VPC)]. NPAF was defined as AF episodes lasting ≥7 days. If sinus rhythm (SR) could not be restored by cardioversion prior to electroanatomic mapping, those NPAF patients were excluded from the study. All antiarrhythmic medications were discontinued for a minimum of 5 half-lives, and amiodarone was ceased for a duration of 2 months prior to catheter ablation. Patient demographics, clinical characteristics, and medications details were obtained from electronic medical records.

This study was approved by the ethics committee of the Third People's Hospital of Chengdu and conducted in compliance with the ethical principles of the Declaration of Helsinki. All patients provided written informed consent.

### The LGE-MRI study

The aim of the LGE-MRI study was to quantify the extent of fibrosis in the LA and LAA. Patients with NPAF underwent LGE-MRI of the LA and LAA using a 3-Tesla scanner (Siemens Skyra, Germany) and a 32-channel cardiac coil within 1 week before the electrophysiological assessment and/or ablation procedure. All patients were in atrial fibrillation (AF) during scanning.

The specific scanning protocol employed a 3D inversion recovery gradient-echo sequence, combined with respiratory navigation and electrocardiogram gating, and was performed 20 min after intravenous administration of gadolinium contrast agent (Gadobutrol, 0.2 mmol/kg). Scanning parameters were as follows: field of view, 350–390 mm; echo time (TE), 1.3–1.6 ms; repetition time (TR), 700–870 ms; inversion time, 310–350 ms; in-plane resolution, 1.37 × 1.37 mm; slice thickness, 1.5 mm; and voxel size, 1.25 × 1.25 × 2.5 mm (reconstructed dimensions: 0.625 × 0.625 × 1.25 mm). Additional typical acquisition parameters included: TR/TE, 5.5/3.0 ms; flip angle, 25°; in-plane resolution, 1.25 × 1.25 mm; and slice thickness, 2.5 mm (reconstructed dimensions: 0.625 × 0.625 × 1.25 mm).

For image post-processing, LGE CMR images were analyzed and segmented using MASS imaging software (Medis Medical Imaging Systems, Leiden, Netherlands). First, raw LGE CMR data were imported into the MASS software. The endocardial and epicardial contours of the LA were then manually delineated on each of the 96 cardiac MRI slices. Based on these segmentation results, the software automatically generated a 3D model of the LA. Myocardial tissue was classified by signal intensity ratio (SIR) as follows (15): healthy atrial myocardium (SIR < 1.2), interstitial fibrosis (SIR: 1.2–1.32), and dense scarring (SIR > 1.32).

### Electrophysiology mapping and ablation procedure

In patients with NPAF, pre-procedure computerized tomography angiography images of the LA were obtained, and transesophageal echocardiography was performed within 48 h prior to the study to rule out left atrial thrombus. Catheter ablation procedures were performed under general anesthesia and muscular paralysis with hemodynamic and electrocardiographic monitoring. A decapolar coronary sinus catheter (2-5-2 mm interelectrode spacing, P-curve, Biosense Webster) was positioned via femoral venous approach under fluoroscopic guidance. Intravenous heparin was administered to maintain an activated clotting time of 300–350 s. Subsequently, LA access was obtained by transseptal puncture, through which a 20-polar mapping catheter (2 mm center-to-center spacing, Pentaray, Biosense Webster) was advanced to create the electroanatomic map. After mapping, a 3.5 mm tip irrigated ablation catheter (1-6-2 mm interelectrode spacing, ThermoCool SmartTouch, Biosense Webster) was advanced to perform ablation in the NPAF study group.

Intracardiac electrograms (EGMs) were recorded using an electrophysiological recording system (WorkMate Claris System, One St. Jude Medical, St. Paul, MN, USA) coupled with a three-dimensional electrophysiological navigating system (CARTO 3 system, Biosense Webster, Diamond Bar, CA, USA). EGM recording was filtered at 30–500 Hz. A detailed fast anatomic map (FAM) was performed with a distribution of points using a fill threshold of 15 mm and an internal point filter was set to limit electric potential data acquisition to within 5 mm from the geometrical surface. The respiration gating method, which ensured the acquisition of anatomic points limited to the end period of expiration during mechanical ventilation, was adopted to minimize the breath related mapping shifts. The CONFIDENSE module (Biosense Webster) was used for tissue proximity detection and to assess adequate tissue-catheter contact of each acquired point during FAM. All mappings were performed by a single, highly experienced electrophysiologist, who implemented rigorous quality control both during real-time data acquisition and offline post-processing.

The following steps were performed to assess bipolar voltage in AF and SR in the NPAF group:

1. In patients presenting in AF, cardioversion was performed to obtain the first electroanatomic map in SR.
2. AF was induced using atrial burst stimuli from the distal or mid-coronary sinus at a cycle of 250–180 ms. Following a 5 min waiting period for rhythm stabilization, voltage mapping was conducted to create an electroanatomic map in AF.
3. Geometries of LA and LAA were created separately using Pentaray high density mapping catheter.
4. The LA geometrical surface was subdivided into 6 anatomical regions: anterior, posterior, roof, inferior, lateral, and septal (16).
5. Points within pulmonary veins (PVs) were excluded from the final analysis.

In consideration of temporal EGM amplitude variability during acquisition, each point was selected by recording a maximal peak-to-peak bipolar voltage amplitude value of local atrial EGM within window of interest during 10 consecutive QRS complexes (except atrial EGMs during each QRS duration). EGMs during premature beats were excluded. A goal of voltage mapping density was set to >200 points in LAA region and >500 points in LA.

In the control group, ablation of accessory pathway or VPC was performed after electroanatomic mapping of the LA and LAA in SR using the same technique as above for NPAF in SR.

### Definitions

The bipolar voltage of dense scar was defined using the cut off value of 0.1 mV. Conventionally, any point with a bipolar voltage ≤ 0.5 mV during SR was classified as a low voltage point. LVA were defined as sites of ≥3 adjacent low voltage points, which were <5 mm apart. For the individualized LVA definition: sites with bipolar voltage ≤ the normalized LVA detection threshold were designated as abnormal substrate. The borders of LVAs were automatically reconstructed in 3D using the CARTO® VOLTAGE VISITAG™ module.

### Statistical analysis

Categorical data were presented as counts and percentages (%) and the comparison between two groups was assessed using the appropriate chi-square test. Continuous data were depicted as either the mean with standard deviation or the median with interquartile range (IQR), and the comparison between two groups was evaluated using the *t*-test or Mann–Whitney *U*-test, respectively. Linear regression analysis was used to evaluate the relationships in LA and LAA voltages. The relationships between LA and LAA voltages with SR and AF were assessed using Spearman correlation coefficients. A two-tailed *p*-value less than 0.05 was considered statistically significant. All statistical analyses were performed with either SPSS statistics version 26.0 (IBM Corporation, Chicago, IL, USA) or GraphPad Prism software version 9.0 (GraphPad Software, CA, USA).

## Results

### Baseline characteristics

The baseline characteristics of all study participants are presented in Table 1. Compared to patients without AF, those with NPAF had larger LA, were older, male, and exhibited a higher prevalence of hypertension and coronary heart disease.

**Table 1:** Baseline characteristics.

Variables	Control	NPAF (*n* = 40)			*P* value*	*P* value**
		Total	Persistent AF	Long-standing persistent AF		
	(*n* = 42)		(*n* = 24)	(*n* = 16)		
Age, years	54.10 ± 16.48	64.15 ± 10.50	64.42 ± 11.67	63.75 ± 8.80	0.001	0.194
Sex, male, *n* (%)	19 (45.2)	28 (70.0)	17	11	0.023	0.888
BMI	23.11 ± 3.06	25.13 ± 3.60	25.12 ± 4.02	25.13 ± 2.96	0.008	0.277
Smoking, *n* (%)	11 (26.2)	15 (37.5)	11 (45.8)	4 (25)	0.271	0.182
Congestive heart failure, *n* (%)	1 (2.4)	4 (10.0)	2 (8.3)	2 (12.5)	0.15	0.667
Hypertension, *n* (%)	7 (16.7)	21 (52.5)	11 (45.8)	10 (62.5)	0.001	0.301
Diabetes mellitus, *n* (%)	6 (14.3)	4 (10.0)	4 (16.7)	0	0.553	0.085
Coronary heart disease, *n* (%)	1 (2.4)	12 (30.0)	8 (33.3)	4 (25)	0.001	0.573
Dyslipidemia, *n* (%)	6 (14.3)	4 (10.0)	3 (12.5)	1 (6.25)	0.553	0.519
Obstructive sleep apnea, *n* (%)	0 (0)	2 (5.0)	2 (8.3)	0	0.142	0.236
Prior stroke, *n* (%)	1 (2.4)	4 (10.0)	3 (12.5)	1 (6.25)	0.150	0.519
CHA_2_DS_2_-VASC score	-	2.42 ± 1.30	2.42 ± 1.21	2.44 ± 1.46	-	0.229
BNP (pg/ml)	96.5 (35, 121.98)	94.6 (49.6, 341.1)	154.75 (67, 98.03)	164.55 (97.63, 320.08)	0.049	0.023
eGFR (ml/min*1.73 m^2^)	98.11 ± 20.36	90.98 ± 20.22	92.19 ± 19.14	89.16 ± 22.27	0.116	0.771
LAD (mm)	35.43 ± 4.62	42.95 ± 5.25	42.13 ± 5.21	44.19 ± 5.23	0.000	0.705
LVDd (mm)	45 (41.75, 48)	47 (44, 50)	47 (43, 50)	47 (44.25, 50)	0.166	0.793
LVEF (%)	60 (58, 63.25)	58.5 (57, 62)	58.5 (56.25, 62)	58.5 (57, 61.75)	0.000	0.883
Time since AF onset, day	-	365 (30, 1,095)	60 (10, 333.75)	1,642.5 (730, 3,011.25)	-	<0.001

The baseline characteristics of the individuals with or without NPAF.

NPAF, non-paroxysmal atrial fibrillation; BMI, body mass index; BNP, brain natriuretic peptide; eGFR, estimated glomerular filtration rate; LAD, left atrial diameter; LVDd, left ventricular diastolic dimension; LVEF, left ventricular ejection fraction.

* *P* value, Control vs. NPAF.

** *P* value, Persistent AF vs. Long-standing AF.

### Electroanatomic mapping and bipolar voltage map

During SR, a mean of 422 ± 185 and 498 ± 372 bipolar voltage points per patient were analyzed in the LA for the control and NPAF group, respectively; An average of 276 ± 139 and 167 ± 67 bipolar voltage points per patient were analyzed in the LAA for the two groups, respectively.

**Figure 1:**
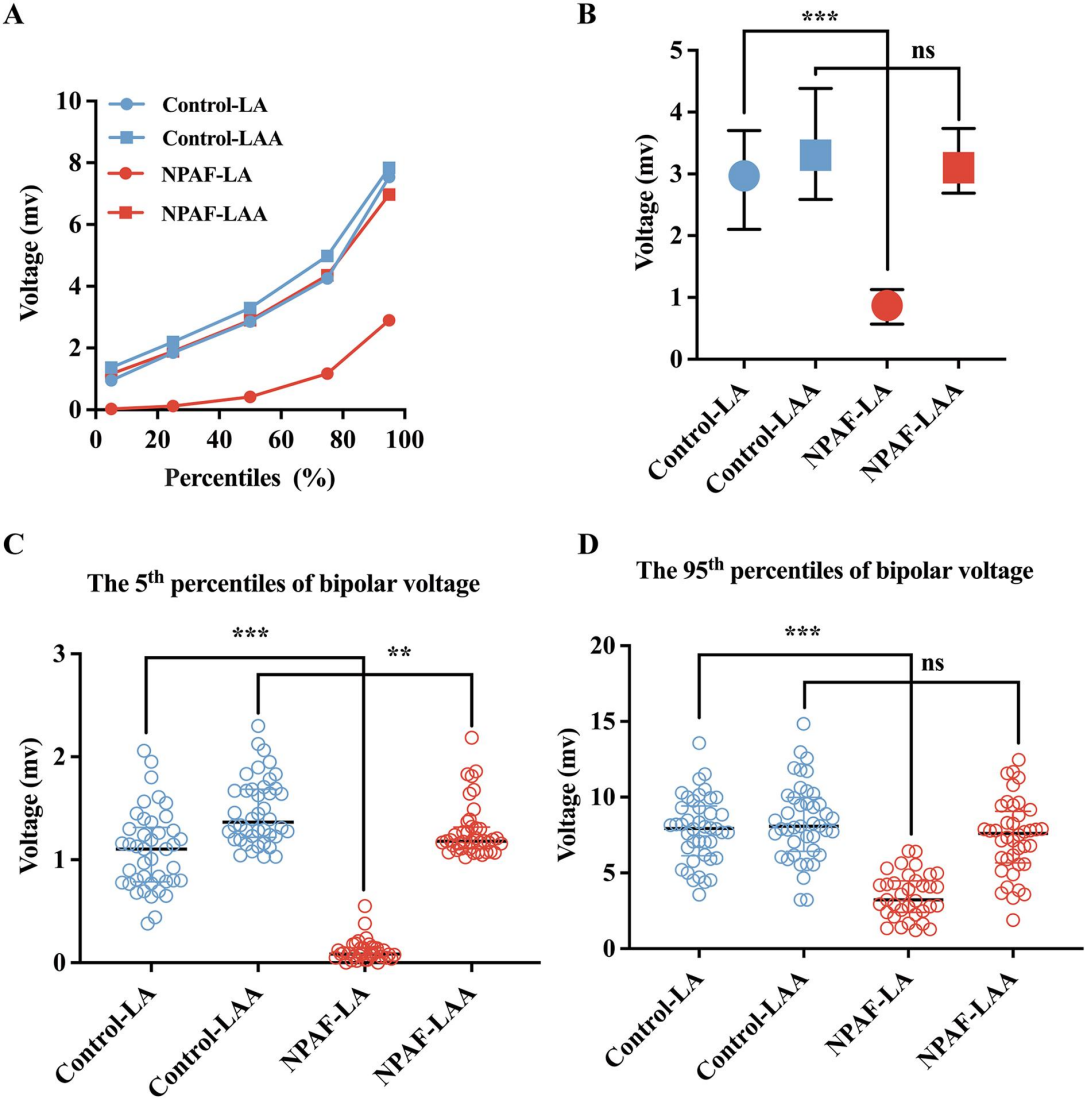
Bipolar voltage comparisons between the LA and LAA. **(A)** The 5th, 25th, 50th, 75th, and 95th percentiles of the bipolar voltage for the LA and LAA in the Control group and NPAF group obtained during SR. **(B)** Voltage for the LA and LAA in the Control group and NPAF group. **(C)** The 5th percentile of voltage in the LA and LAA during SR. **(D)** The 95th percentile of voltage in the LA and LAA during SR. NPAF, non-paroxysmal atrial fibrillation; LA, left atrium; LAA, left atrial appendage; SR, sinus rhythm. ***, *P* < 0.001; **, *P* < 0.01; ns, no significance. Data are expressed as the median and interquartile range.

Compared to patients without NPAF, the 5th, 25th, 50th, 75th, and 95th percentiles of bipolar voltage in the LA were significantly lower in patients with NPAF (Figure 1A). The bipolar voltage in the LAA was significantly higher than the LA in patients with NPAF, but not in the control group (Figure 1A). The median bipolar voltage in the LA was significantly lower in patients with NPAF when compared to the control group [2.970 (1.599) vs. 0.870 (0.560), *P* < 0.001; Figure 1B]. However, there was no significant difference in LAA bipolar voltage between the two groups [3.303 (1.796) vs. 3.100 (1.045), *P* > 0.05; Figure 1B]. The 5th and 95th percentiles of bipolar voltage in the LA were significantly lower in patients with NPAF compared to the control group [1.103 (0.529) vs. 0.085 (0.096), *P* < 0.001, Figure 1C; 7.937 (3.282) vs. 3.232 (2.087), *P* < 0.001, Figure 1D]. No significant difference was observed in the 95th percentile of bipolar voltage in the LAA (V_LAA95%_) between the two groups [8.089 (3.571) vs. 7.604 (3.404), *P* > 0.05; Figure 1D]. The V_LAA95%_ in patients without NPAF conformed to a normal distribution. We used the V_LAA95%_ as a benchmark given the relative similarity in patients with and without AF.

### Defining patient-specific normalized low voltage threshold

**Figure 2:**
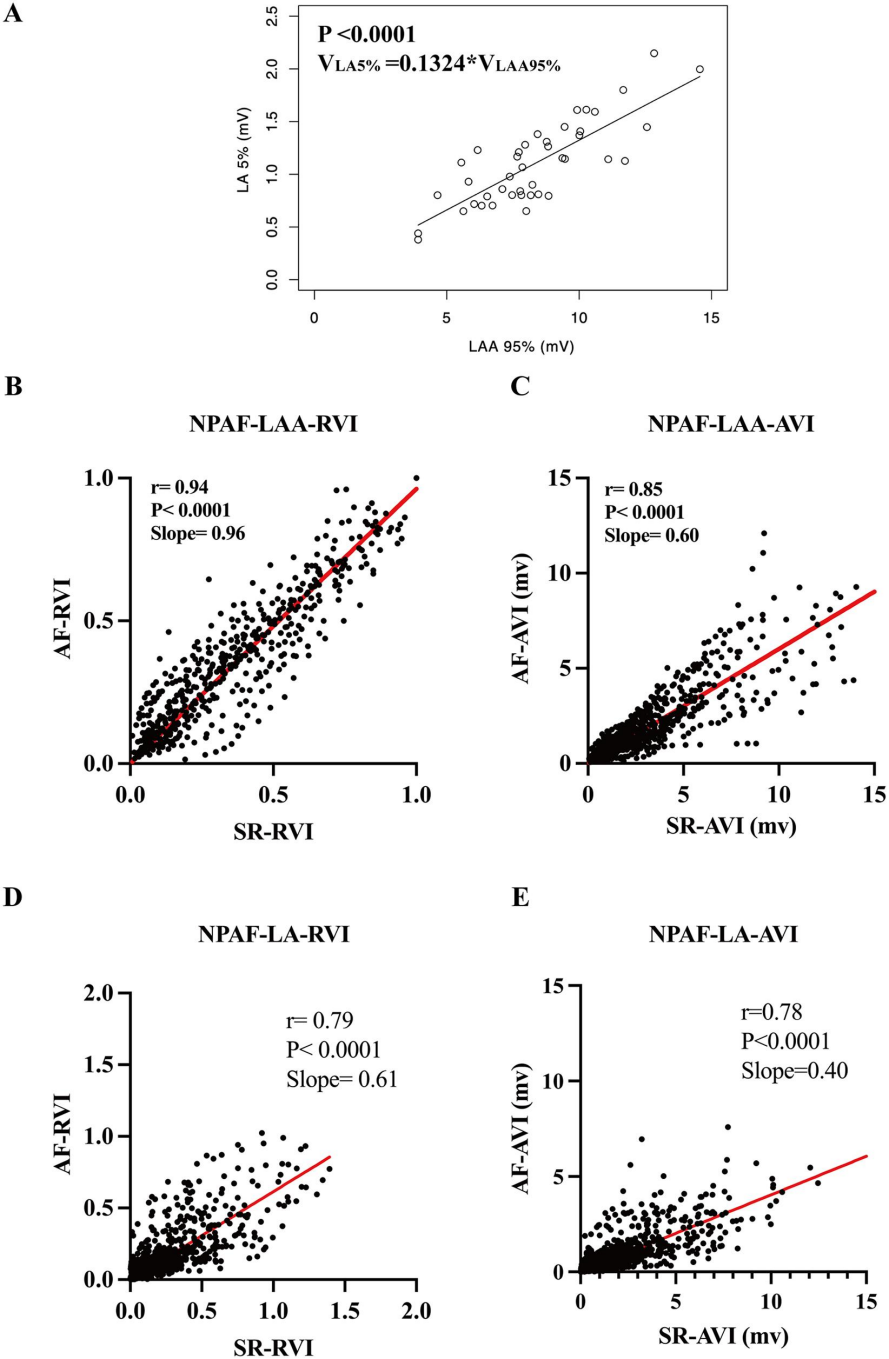
Establishment and validation of a normalized relative voltage index equation. **(A)** The regression equation of the 5th percentile of voltage in the LA (V_LA 5%_) and the 95th percentile of voltage in the LAA (V_LAA 95%_) used in the normalized RVI threshold equation. Note correlation of the RVI in the LAA **(B)** and the LA **(D)** in SR and AF rhythm. Comparisons made to the AVI in the LAA and LA in SR and AF rhythm are shown in **(C,E)**, respectively. RVI, relative voltage index; AVI, absolute voltage index. Other abbreviations as per Figure 1.

In previous studies, in control patients without AF, the 5th percentile of all LA mapping points (V_LA5%_) was designated as a bipolar voltage threshold indicating abnormal atrial substrate (17, 18). Thus, a point with a voltage lower than V_LA5%_ was defined as low voltage. In the present study, a strong linear correlation between V_LA5%_ and V_LAA95%_ (*P* < 0.0001, V_LA5%_ = 0.1324 × V_LAA95%_, Figure 2A) was observed in the control group. The ratio of each point's voltage to V_LAA95%_ in each patient was termed the ***relative voltage index*** (RVI). When adjusting each point's voltage in the LAA and LA by V_LAaA95%_ and expressing them as RVI, the scattergrams revealed a notably stronger linear correlation of the electrogram amplitudes between SR and AF (Figures 2B,D). However, when directly using the absolute voltage of each point (AVI) in the LAA and LA, the scattergrams displayed a significant but weaker linear correlation of the bipolar voltage between SR and AF (Figures 2C,E).

**Figure 3:**
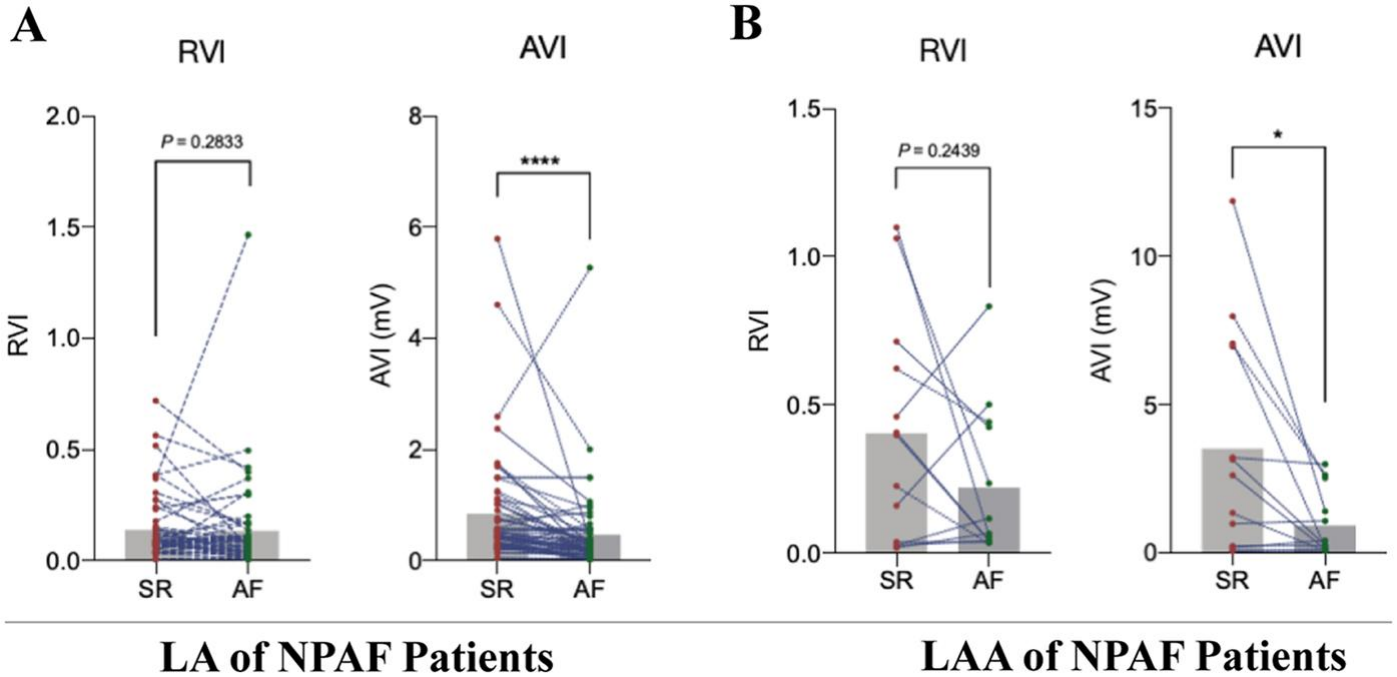
Establishing the applicability of the relative voltage index to patients with Non-paroxysmal atrial fibrillation. **(A)** Pairwise *t*-test of mapping results in LA for sinus rhythm (SR) and atrial fibrillation (AF) under relative voltage index (RVI) and absolute voltage index (AVI) in patients with non-paroxysmal atrial fibrillation (NPAF), *n* = 40. **(B)** Pairwise *t*-test of mapping results in LAA for SR and AF under RVI and AVI in patients with NPAF, *n* = 40.

Paired *t*-tests revealed that the absolute voltage index (AVI) values of the LA and LAA exhibited statistically significant differences in the same patient between SR and atrial AF states. In contrast, the RVI showed no statistically significant differences between SR and AF states in either the LA or LAA regions (Figures 3A,B). These results suggest that the correlation between V_LAA95%_ and V_LA5%_ established in control patients could applicability to patients with NPAF. These results suggest that the correlation between V_LAA95%_ and V_LA5%_ established in control patients could be applicable to patients with NPAF.

### Determination of V_LA5%_ in NPAF patients

In the control group, the RVI of V_LA5%_ to V_LAA95%_ approaches a constant as indicated by the regression equation mentioned above. This constant was identified as the standard relative voltage index (SRVI) in this study, serving as the threshold value for the RVI criterion in defining LVA. The V_LA5%_ of normal LA is defined as the low voltage threshold value, and any point with a voltage lower than “normal” V_LA5%_ should be classified as a low voltage point for that patient. However, the “normal” V_LA5%_ in a patient with NPAF is unavailable due to diseased myocardium. Herein, the “normal” V_LA5%_ for each NPAF patient can be calculated with the equation: V_LA5%_ = 0.1324 × V_LAA95%_.

### LVA determined by different threshold settings to match LGE-MRI

**Figure 4:**
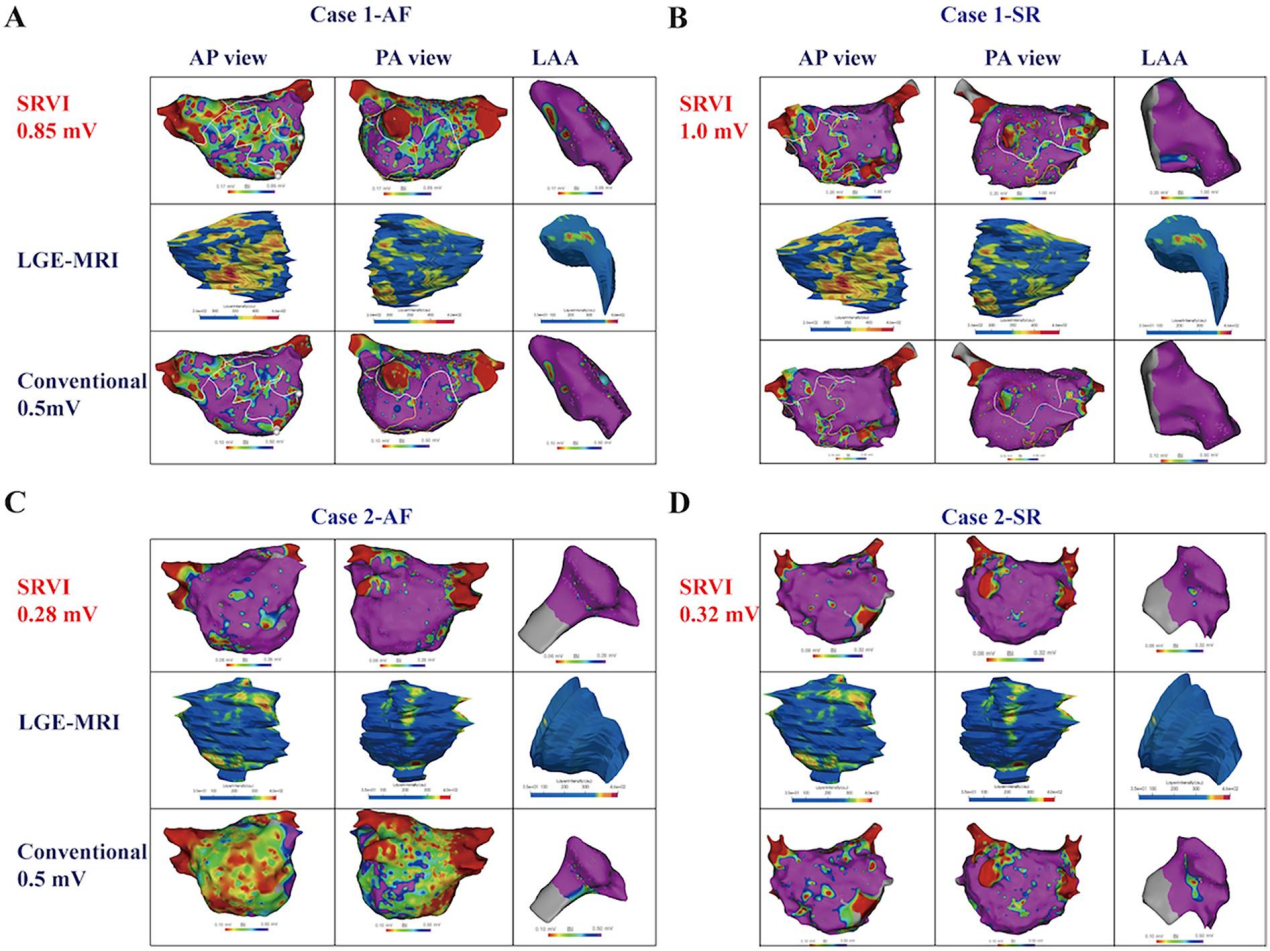
Comparison between SRVI defined LVA and conventional voltage threshold in NPAF patients. Case 1. Comparison between LVA defined by SRVI [<0.85 mV in AF **(A)** and <1.0 mV in SR **(B)**] and the conventional low voltage threshold of <0.5 mV and correlation with LGE on cardiac MRI. Note the visual underestimation of fibrosis in the conventional voltage threshold as compared to LGE especially in AF whereas the SRVI provides a more accurate representation. Case 2. Comparison between LVA defined by SRVI [<0.28 mV in AF **(C)** and <0.32 mV in SR **(D)**] and the conventional low voltage threshold of <0.5 mV and correlation with LGE on cardiac MRI. Note the visual overestimation of fibrosis based on conventional voltage thresholds especially in AF whereas the SRVI provides a more accurate representation. LGE-MRI, late gadolinium enhancement MRI; SRVI, standard relative voltage index. Other abbreviations as per Figure 1.

Following electroanatomic mapping of the LA and LAA, the V_LAA95%_ is determined. Utilizing the formula V_LA5%_ = 0.1324 × V_LAA95%_, the patient-specific V_LA5%_ is calculated, which functions as the normalized threshold for identifying LVA within the LA in patients with NPAF. In the cohort of 40 NPAF patients, the SRVI-guided substrate mapping can be visually appreciated as a better match to the fibrotic regions of LGE in both rhythms as compared to the conventional voltage threshold for LVA (Table 2). Two retrospective cases of NPAF were presented to compare the distributions of LVA defined by SRVI and conventional threshold of 0.5 mV with fibrosis regions of LGE-MRI in both AF and SR (Figure 4). The V_LA5%_ for Case 1 was 0.85 mV in AF and 1.0 mV in SR (Figures 4A,B). For Case 2, the V_LA5%_ was 0.28 mV in AF and 0.32 mV in SR (Figures 4C,D). In comparison to the conventional LVA threshold of 0.5 mV, LVA in both the anterior and posterior walls of the LA in NPAF patients during AF, as defined by the normalized criteria-guided substrate mapping, exhibited a stronger correlation with the fibrotic regions of LGE.

**Table 2:** Comparing the efficacy of SRVI and AVI in detecting left atrial LVA in NPAF.

Imaging view	SRVI	Conventional setting (0.5 mV)	*P* value
	(*n* = 40)		
AP view, *n* (%)	26 (65)	15 (37.5)	0.014
PA view, *n* (%)	23 (57.5)	13 (32.5)	0.025

Visual comparison of the performance of the SRVI and the conventional voltage threshold (<0.5 mV) in detecting LVA based on MRI-defined fibrosis. SRVI, standard relative voltage index; LVA, low voltage areas. AP (anterior to posterior) and PA (posterior to anterior) views reference the imaging angle used to visualize the left atrium.

### The distribution and extent of LVA determined by different threshold settings

A low voltage index was employed to define the heterogeneity of the bipolar voltage amplitude by computing the coefficient of variance (standard deviation/mean value) of the voltage of all points (18). The regional and overall differences in the low voltage index based on SRVI and conventional LVA detection threshold of 0.5 mV in both rhythms are summarized in Table 3. During both rhythms, the low voltage index was significantly higher in AF than SR across all LA segments, regardless of the threshold settings. The correlation between the low voltage index in both rhythms, as defined by the normalized RVI-guided substrate mapping, was superior to the conventional LVA detection threshold value of 0.5 mV.

**Table 3:** The extent of LVA detected by different threshold settings.

Anatomical region	SRVI		Conventional setting (0.5 mV)	
	SR (*n* = 40)	AF (*n* = 40)	SR (*n* = 40)	AF (*n* = 40)
LAA (%)	26.09 ± 12.95	23.91 ± 15.44	15.44 ± 12.27	20.06 ± 15.33
Anterior (%)	62.39 ± 19.03	71.84 ± 25.20	37.92 ± 23.32	63.04 ± 27.39
Inferior (%)	50.05 ± 21.10	73.10 ± 19.83	29.41 ± 18.87	61.95 ± 23.60
Septal (%)	65.36 ± 18.17	71.92 ± 22.14	41.07 ± 19.23	64.21 ± 24.11
Lateral (%)	40.63 ± 24.49	63.77 ± 23.72	31.44 ± 24.23	56.21 ± 26.15
Posterior (%)	53.55 ± 23.51	68.35 ± 21.45	34.28 ± 25.14	59.14 ± 25.92
Roof (%)	53.51 ± 27.83	75.07 ± 24.82	35.07 ± 28.54	63.03 ± 31.46
LA (%)	54.11 ± 18.70	70.33 ± 17.34	34.74 ± 19.50	61.10 ± 23.37

LVA, low voltage areas; SR, sinus rhythm; AF, atrial fibrillation; SRVI, standard relative voltage index; LAA, left atrial appendage; LA, left atrium.

## Discussion

To the best of our knowledge, this study is the first to evaluate a novel patient-specific definition of LVA using a simplified equation in NPAF.

The main findings are as follows:

1. A novel patient-specific SRVI threshold for low voltage outperforms conventional voltage thresholds for LVA and can be used in SR or AF.
2. Unlike LA voltage, LAA bipolar voltage amplitudes are not significantly different between patients with normal LA and patients with NPAF.
3. There is a strong linear correlation between V_LA5%_ and V_LAA95%_ in the normal LA.

### Significance of voltage differences by electroanatomic mapping

The origin of low amplitude atrial electrograms can be multifactorial but atrial fibrosis is considered a primary structural cause (19). Bipolar voltage mapping is used as a surrogate for identification of histopathological LA fibrosis despite inherent limitations (8–10, 13, 20). Studies have demonstrated a strong correlation between bipolar voltage mapping and LGE-MRI, which is considered the gold standard for identification of LA fibrosis (5, 6). However, there is no unified criteria for detecting LVA in patients with NPAF across different rhythms and with different recording electrodes. Voltage is influenced by the extent of LA fibrosis which relates to clinical factors such as age, LA size, comorbidities, and duration of AF (18, 21, 22). Voltage is also affected by the rhythm mapped, regional wall thickness, and the duration of acquisition (21–25). Using a universal absolute voltage cut-off to define LVA in NPAF disregards the variable factors that may influence bipolar voltage in each individual case. Our study refines the definition of LVA in NPAF to account for a population with heterogeneous fibrotic burden and distribution.

### Validation of voltage thresholds for defining LVA

Voltage thresholds to identify low voltage have been classified based on the 95th percentile of voltage in patients with and without AF (17, 18, 26). Multiple thresholds have been reported for identifying LVA across different phenotypes of AF (PAF and NPAF) during both AF and SR. A universal constant, such as 0.5 mV and 0.4 mV, has been established as the absolute threshold for identifying LVA in AF patients (18, 27–29). Fibrotic burden by MRI remains the gold standard for identification of substrate. Low voltage thresholds have limited specificity and sensitivity for detection of fibrosis by MRI but appear to be more predictive when mapping in AF as compared to SR (25). Using the LAA voltage as a reference, the equation for determining a normalized LVA threshold value in the LA for NPAF is highlighted in the current study (V_LA5%_ = 0.1324 × V_LAA95%_). We have validated this voltage index with gold standard MRI in our study and provided a more accurate representation of histopathological fibrosis during electroanatomic mapping.

### Use of the LAA in normalizing bipolar voltage

The observed voltage discrepancy between the LAA as compared to the LA in NPAF is consistent with the study by Yagishita et al. noting that the highest voltages were observed in the LAA in patients with PAF and NPAF in SR and AF (24). Our study expands the analysis to patients with normal LA (control group—no PAF or NPAF). We found no significant difference in the distribution of LAA voltage between the control group and NPAF group during SR, especially for the V_LAA95%_. Notably, we extended this observation by including a control cohort (patients with normal LA, no PAF/NPAF) and demonstrating selective preservation of LAA voltage in NPAF: while all LA voltage percentiles (5th, 25th, 50th, 75th, 95th) were significantly lower in NPAF vs. controls, LAA voltage—including V_LAA95%_—has no significant difference between groups (median LAA voltage: 3.303 [1.796] vs. 3.100 [1.045], *P* > 0.05; V_LAA95%_: 8.089 [3.571] vs. 7.604 [3.404], *P* > 0.05) during SR. This stability indicates the electrophysiological characteristics of LAA is spared from the structural remodeling that drives LA voltage attenuation in NPAF.

Consistent with prior studies in NPAF, which have demonstrated a lower LGE burden in the LAA compared with the LA, this reduced fibrotic involvement may explain the LAA's electrophysiological stability (18, 25). Factors that may make the LAA less susceptible to fibrosis include a lower proportion of interstitial cells, robust arterial supply, and enhanced wall thickness (30, 31). Intuitively, the LAA voltage (V_LAA95%_) would be the most appropriate benchmark to establish a standard RVI for detection of LVA in each case of NPAF.

### Rhythm-dependent low voltage assessment

Studies exploring the relationship between bipolar voltage in SR and AF have had mixed results. Linear correlations have been shown but the influence of other electrophysiological factors such as electrogram fractionation may play a significant role in voltage differences between SR and AF (22, 24). Moisés et al. suggested that bipolar voltage thresholds for LVA should be adjusted depending on the rhythm and found that employing a modified lower detection threshold for LVA during mapping might produce valid and reliable results for identifying regions with a voltage of <0.5 mV in SR (23). There is a paucity of data on the ideal voltage threshold for identifying LVA in AF, however. Differences due to wavefront collision, spiral waves, and critical repolarization/depolarization mass may result in significant voltage threshold changes in SR vs. AF. In one study, the LVA threshold during AF was half of the standard threshold used in SR (23). Variability in AF cycle length, location of recording, and substrate heterogeneity may limit the reproducibility of LVA thresholds when used during AF. The personalized RVI that we have proposed in this study simplifies the assessment of LVA during electroanatomic mapping. Our findings suggest that the RVI can be used for LVA assessment in a rhythm-independent fashion.

### Clinical implications

A simplified and tailored approach to LVA threshold assessment with the RVI enhances the identification of atrial substrate in each individual patient without the influence of the type of rhythm. This may lead to improved efficiency of mapping and a better understanding of the underlying pathology for prognostication, especially if advanced cardiac imaging (LGE-MRI) is unavailable or undesired. Whether the use of RVI can more accurately identify and improve outcomes after voltage-based extra-pulmonary vein substrate modification during AF catheter ablation is unknown and deserves further investigation.

### Study limitations

There were several limitations in our study. Patients with AF at the beginning of the case were cardioverted to SR for the first mapping study. This may not be achievable in all cases of NPAF. Although our LA mapping density meets current high-density standards, we acknowledge it may be relatively low depending on the contemporary mapping system used; future studies employing ultra-high-density mapping could potentially achieve finer substrate characterization. Voltage measurements in SR may be underestimated due stunned myocardium after cardioversion. Apart from comparing bipolar voltage, we did not analyze other electrogram characteristics, such as unipolar voltage, cycle length, fractionated electrograms, and rotational activity across varied voltage settings and rhythms. We also did not explore the influence of additional wavefronts during sinus rhythm on voltage amplitudes. Additionally, prior studies have shown that patients with left-sided accessory pathways [which constitute the majority of Wolff-Parkinson-White (WPW) syndrome cases] exhibit a significantly higher prevalence of AF compared to the general population, with paroxysmal AF occurring in up to one-third of WPW patients (32, 33). This association indicates that left-sided accessory pathways are linked to an atrial substrate with heightened AF susceptibility and the selection of such patients as the control group may introduce potential confounding.

## Conclusions

A patient-tailored, rhythm-independent, low voltage threshold index can be obtained using a simplified equation and provides a more accurate representation of LVA in NPAF than universal voltage thresholds.

## Data Availability

The original contributions presented in the study are included in the article/Supplementary Material, further inquiries can be directed to the corresponding authors.

## References

[1] Stewart S Hart CL Hole DJ McMurray JJ. A population-based study of the long-term risks associated with atrial fibrillation: 20-year follow-up of the renfrew/paisley study. Am J Med. (2002) 113:359–64. 10.1016/S0002-9343(02)01236-6 12401529

[2] Lloyd-Jones DM Wang TJ Leip EP Larson MG Levy D Vasan RS Lifetime risk for development of atrial fibrillation: the framingham heart study. Circulation. (2004) 110:1042–6. 10.1161/01.CIR.0000140263.20897.42 15313941

[3] Dzeshka MS Lip GY Snezhitskiy V Shantsila E. Cardiac fibrosis in patients with atrial fibrillation: mechanisms and clinical implications. J Am Coll Cardiol. (2015) 66:943–59. 10.1016/j.jacc.2015.06.1313 26293766

[4] Platonov PG Mitrofanova LB Orshanskaya V Ho SY. Structural abnormalities in atrial walls are associated with presence and persistency of atrial fibrillation but not with age. J Am Coll Cardiol. (2011) 58:2225–32. 10.1016/j.jacc.2011.05.061 22078429

[5] Mahnkopf C Badger TJ Burgon NS Daccarett M Haslam TS Badger CT Evaluation of the left atrial substrate in patients with lone atrial fibrillation using delayed-enhanced MRI: implications for disease progression and response to catheter ablation. Heart Rhythm. (2010) 7:1475–81. 10.1016/j.hrthm.2010.06.030 20601148 PMC3106345

[6] Oakes RS Badger TJ Kholmovski EG Akoum N Burgon NS Fish EN Detection and quantification of left atrial structural remodeling with delayed-enhancement magnetic resonance imaging in patients with atrial fibrillation. Circulation. (2009) 119:1758–67. 10.1161/CIRCULATIONAHA.108.811877 19307477 PMC2725019

[7] Bijvoet GP Nies H Holtackers RJ Linz D Adriaans BP Nijveldt R Correlation between cardiac MRI and voltage mapping in evaluating atrial fibrosis: a systematic review. Radiol Cardiothorac Imaging. (2022) 4:e220061. 10.1148/ryct.220061 36339060 PMC9627236

[8] Junarta J Siddiqui MU Riley JM Dikdan SJ Patel A Frisch DR. Low-voltage area substrate modification for atrial fibrillation ablation: a systematic review and meta-analysis of clinical trials. Europace. (2022) 24:1585–98. 10.1093/europace/euac089 35696286

[9] Siebermair J Kholmovski EG Marrouche N. Assessment of left atrial fibrosis by late gadolinium enhancement magnetic resonance imaging: methodology and clinical implications. JACC Clin Electrophysiol. (2017) 3:791–802. 10.1016/j.jacep.2017.07.004 29759774

[10] Liu W Li S Han B. It is necessary to re-understand the low-voltage area in atrial fibrillation patients. Front Cardiovasc Med. (2022) 9:919873. 10.3389/fcvm.2022.919873 35783829 PMC9247271

[11] Yamaguchi T Otsubo T Takahashi Y Nakashima K Fukui A Hirota K Atrial structural remodeling in patients with atrial fibrillation is a diffuse fibrotic process: evidence from high-density voltage mapping and atrial biopsy. J Am Heart Assoc. (2022) 11:e024521. 10.1161/JAHA.121.024521 35261287 PMC9075313

[12] Caixal G Alarcón F Althoff TF Nuñez-Garcia M Benito EM Borràs R Accuracy of left atrial fibrosis detection with cardiac magnetic resonance: correlation of late gadolinium enhancement with endocardial voltage and conduction velocity. Europace. (2021) 23:380–8. 10.1093/europace/euaa313 33227129

[13] Verma A Wazni OM Marrouche NF Martin DO Kilicaslan F Minor S Pre-existent left atrial scarring in patients undergoing pulmonary vein antrum isolation: an independent predictor of procedural failure. J Am Coll Cardiol. (2005) 45:285–92. 10.1016/j.jacc.2004.10.035 15653029

[14] Moustafa A Karim S Kahaly O Elzanaty A Meenakshisundaram C Abi-Saleh B Low voltage area guided substrate modification in nonparoxysmal atrial fibrillation: a systematic review and meta-analysis. J Cardiovasc Electrophysiol. (2023) 34:455–64. 10.1111/jce.15764 36453469

[15] Benito EM Carlosena-Remirez A Guasch E Prat-González S Perea RJ Figueras R Left atrial fibrosis quantification by late gadolinium-enhanced magnetic resonance: a new method to standardize the thresholds for reproducibility. Europace. (2017) 19:1272–9. 10.1093/europace/euw219 27940935

[16] Huang W Sun H Xiong S Luo Y Tang Y Zhang Z Sex-related differences in left atrial substrate among patients with atrial fibrillation: evidence from high-density voltage mapping. Eur J Med Res. (2024) 29:354. 10.1186/s40001-024-01952-y 38956703 PMC11218306

[17] Saghy L Callans DJ Garcia F Lin D Marchlinski FE Riley M Is there a relationship between complex fractionated atrial electrograms recorded during atrial fibrillation and sinus rhythm fractionation? Heart Rhythm. (2012) 9:181–8. 10.1016/j.hrthm.2011.09.062 21946341

[18] Lin Y Yang B Garcia FC Ju W Zhang F Chen H Comparison of left atrial electrophysiologic abnormalities during sinus rhythm in patients with different type of atrial fibrillation. J Interv Card Electrophysiol. (2014) 39:57–67. 10.1007/s10840-013-9838-y 24113851

[19] Allessie M Ausma J Schotten U. Electrical, contractile and structural remodeling during atrial fibrillation. Cardiovasc Res. (2002) 54:230–46. 10.1016/S0008-6363(02)00258-4 12062329

[20] Marrouche NF Wilber D Hindricks G Jais P Akoum N Marchlinski F Association of atrial tissue fibrosis identified by delayed enhancement MRI and atrial fibrillation catheter ablation: the DECAAF study. Jama. (2014) 311:498–506. 10.1001/jama.2014.3 24496537

[21] Mannion J Hong K Lennon SJ Kenny A Galvin J O'Brien J Comparing left atrial low voltage areas in sinus rhythm and atrial fibrillation using novel automated voltage analysis: a pilot study. Cardiol Res. (2023) 14:268–78. 10.14740/cr1503 37559712 PMC10409550

[22] Masuda M Fujita M Iida O Okamoto S Ishihara T Nanto K Comparison of left atrial voltage between sinus rhythm and atrial fibrillation in association with electrogram waveform. Pacing Clin Electrophysiol. (2017) 40:559–67. 10.1111/pace.13051 28211132

[23] Rodríguez-Mañero M Valderrábano M Baluja A Kreidieh O Martínez-Sande JL García-Seara J Validating left atrial low voltage areas during atrial fibrillation and atrial flutter using multielectrode automated electroanatomic mapping. JACC Clin Electrophysiol. (2018) 4:1541–52. 10.1016/j.jacep.2018.08.015 30573117

[24] Yagishita A De Oliveira S Cakulev I Gimbel JR Sparano D Manyam H Correlation of left atrial voltage distribution between Sinus rhythm and atrial fibrillation: identifying structural remodeling by 3-D electroanatomic mapping irrespective of the rhythm. J Cardiovasc Electrophysiol. (2016) 27:905–12. 10.1111/jce.13002 27135965

[25] Qureshi NA Kim SJ Cantwell CD Afonso VX Bai W Ali RL Voltage during atrial fibrillation is superior to voltage during sinus rhythm in localizing areas of delayed enhancement on magnetic resonance imaging: an assessment of the posterior left atrium in patients with persistent atrial fibrillation. Heart Rhythm. (2019) 16:1357–67. 10.1016/j.hrthm.2019.05.032 31170484 PMC6722483

[26] Kapa S Desjardins B Callans DJ Marchlinski FE Dixit S. Contact electroanatomic mapping derived voltage criteria for characterizing left atrial scar in patients undergoing ablation for atrial fibrillation. J Cardiovasc Electrophysiol. (2014) 25:1044–52. 10.1111/jce.12452 24832482

[27] Yang G Yang B Wei Y Zhang F Ju W Chen H Catheter ablation of nonparoxysmal atrial fibrillation using electrophysiologically guided substrate modification during Sinus rhythm after pulmonary vein isolation. Circ Arrhythm Electrophysiol. (2016) 9:e003382. 10.1161/CIRCEP.115.003382 26857907

[28] Yagishita A Gimbel JR De Oliveira S Manyam H Sparano DI Cakulev IV Long-term outcome of left atrial voltage-guided substrate ablation during atrial fibrillation: a novel adjunctive ablation strategy. J Cardiovasc Electrophysiol. (2017) 28:147–55. 10.1111/jce.13122 27862561

[29] Lo LW Tai CT Lin YJ Chang SL Wongcharoen W Chang SH Progressive remodeling of the atrial substrate–a novel finding from consecutive voltage mapping in patients with recurrence of atrial fibrillation after catheter ablation. J Cardiovasc Electrophysiol. (2007) 18:258–65. 10.1111/j.1540-8167.2007.00719.x 17241372

[30] Naksuk N Padmanabhan D Yogeswaran V Asirvatham SJ. Left atrial appendage: embryology, anatomy, physiology, arrhythmia and therapeutic intervention. JACC Clin Electrophysiol. (2016) 2:403–12. 10.1016/j.jacep.2016.06.006 29759858

[31] Spach MS Dolber PC. Relating extracellular potentials and their derivatives to anisotropic propagation at a microscopic level in human cardiac muscle. Evidence for electrical uncoupling of side-to-side fiber connections with increasing age. Circ Res. (1986) 58:356–71. 10.1161/01.RES.58.3.356 3719925

[32] Sharma AD Klein GJ Guiraudon GM Milstein S. Atrial fibrillation in patients with Wolff-Parkinson-White syndrome: incidence after surgical ablation of the accessory pathway. Circulation. (1985) 72:161–9. 10.1161/01.CIR.72.1.161 4006127

[33] Haissaguerre M Fischer B Labbé T Lemétayer P Montserrat P d'Ivernois C Frequency of recurrent atrial fibrillation after catheter ablation of overt accessory pathways. Am J Cardiol. (1992) 69(5):493–7. 10.1016/0002-9149(92)90992-8 1736613

