# Supplementary material for: The relative voltage index: a novel tailored method to identify left atrial low voltage areas in non-paroxysmal AF
Source: Front Cardiovasc Med. 2025 Sep 16;12:1656983. doi: 10.3389/fcvm.2025.1656983 (PMC12479551; doi:10.3389/fcvm.2025.1656983)
Supplement: Supplementary file 1 [file Image1.pdf]

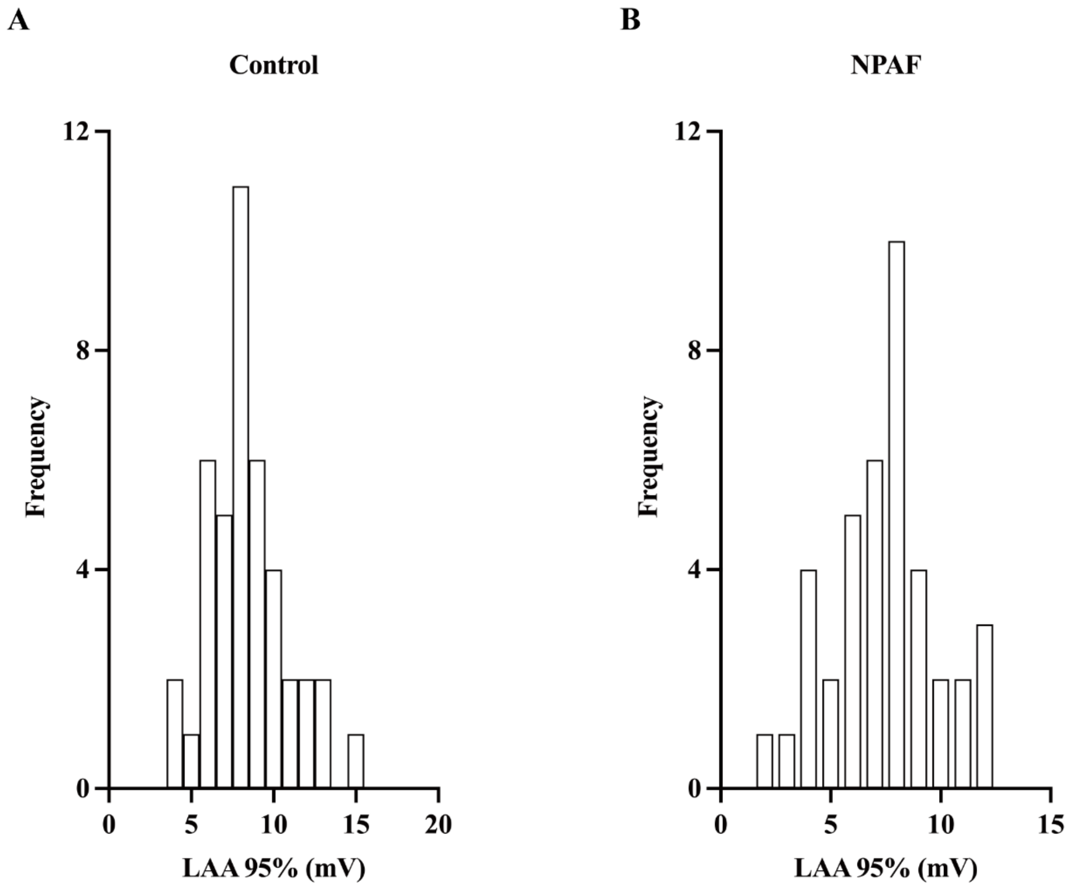

Figure S1 Assessing Normality of the 95th Percentile of Voltages in the LAA

The normality of the 95th percentile of voltages of the LAA in patients from Control group (**A**) and NPAF group (**B**). LAA = left atrial appendage; NPAF = non-paroxysmal atrial fibrillation.
